# Systematic optimization of square-wave electroporation conditions for bovine primary fibroblasts

**DOI:** 10.1186/s12860-020-00254-5

**Published:** 2020-02-28

**Authors:** Iqbal Hyder, Shahin Eghbalsaied, Wilfried A. Kues

**Affiliations:** 1 Institute of Farm Animal Genetics, Friedrich-Loeffler-Institute, 31535 Neustadt, Germany; 2 Department of Veterinary Physiology, NTR College of Veterinary Science, Gannavaram, India; 3 Transgenesis Center of Excellence, Isfahan (Khorasgan) branch, Islamic Azad University, Isfahan, Iran

**Keywords:** Fibroblasts, Electroporation, Transfection efficiency, Square wave pulse

## Abstract

**Background:**

Gene transfer by electroporation is an established method for the non-viral mediated transfection of mammalian cells. Primary cells pose a particular challenge for electroporation-mediated gene transfer, since they are more vulnerable than immortalized cells, and have a limited proliferative capacity. Improving the gene transfer by using square wave electroporation in difficult to transfect cells, like bovine fetal fibroblasts, is a prerequisite for transgenic and further downstream experiments.

**Results:**

Here, bovine fetal fibroblasts were used for square-wave electroporation experiments in which the following parameters were systematically tested: electroporation buffer, electroporation temperature, pulse voltage, pulse duration, pulse number, cuvette type and plasmid DNA amount. For the experiments a commercially available square-wave generator was applied. Post electroporation, the bovine fetal fibroblasts were observed after 24 h for viability and reporter expression. The best results were obtained with a single 10 millisecond square-wave pulse of 400 V using 10 μg supercoiled plasmid DNA and 0.3 × 10^6^ cells in 100 μl of Opti-MEM medium in 4 mm cuvettes. Importantly, the electroporation at room temperature was considerably better than with pre-cooled conditions.

**Conclusions:**

The optimized electroporation conditions will be relevant for gene transfer experiments in bovine fetal fibroblasts to obtain genetically engineered donor cells for somatic cell nuclear transfer and for reprogramming experiments in this species.

## Background

Electroporation is a physical method that can be used for gene delivery characterized by application of brief electric pulses to permeabilize the cell membrane, and thereby facilitating the uptake of negatively charged DNA [1, 2]. The application of a potential difference across a membrane is an effective strategy to form transient pores [3]. In principle, cell membranes act as electrical capacitors and the application of a high-voltage electric field results in a temporary depolarization of a cell membrane and the formation of pores, which allows the entrance of macromolecules. The application of electric pulses is not only used for cell permeabilization in vitro for delivery of micro-and macromolecules, but is also used in vivo for permeabilization of tissues during certain specific treatments against cancers via electrochemotherapy (ECT) where electric pulses are applied to enable entry of non-permeant cytotoxic molecules [4]. The conventional electroporation is done in cuvette-style parallel plate setups, where the cell suspension and molecules to-be-delivered are mixed together in the electroporation buffer between two plate electrodes connected to a generator of high electric voltage, and is called bulk electroporation [3]. Electroporation is viewed as a promising method for intracellular delivery of a wide variety of cargos and being relatively efficient as compared to other methods [3, 5]. Fibroblasts are the most preferred somatic cells in gene transfection studies, since they can be derived either from fetal or adult tissue samples [6]. Many authors previously reported the use of electroporation in bovine fibroblasts and in fibroblastoid cells of other mammals as an efficient method of DNA transfection [7]. Though primary fibroblasts are commonly used cells in many studies, they are considered as difficult to transfect cells [8]. Till date, few data are available describing the optimization of electroporation conditions for bovine fetal fibroblasts (BFFs). Cattle is an economically important livestock [9], and increasingly used as a model species for research in artificial reproduction [10, 11]. The establishment of somatic cell nuclear transfer (SCNT) [12] allowed the generation of transgenic and knock-out cattle via the use of genetically modified fibroblast donor cells [13, 14]. The recently developed designer nuclease (ZNF, TALEN and Crispr/Cas9) were also employed to edit endogenous genes or knock-in genes-of-interest into bovine primary cells, which are subsequently used in animal cloning via SCNT [15–19]. These examples highlight the importance of efficient transfection methods for bovine primary cells.

In principal, two distinct wave forms of a pulse can be generated in a bulk electroporation setting, exponential decay and square wave [20]. Whereas both wave forms were used for electroporation, the latter was proven to be optimal [20] for mammalian cells. Square-wave electroporators represent the most widely used systems, they allow to control both voltage and pulse duration, and can produce rapidly repeating pulses. Several factors play a critical role in optimal transfection during electroporation. These include pulse amplitude, number, duration, interval between multiple pulses, and cuvette type [21, 22]. The most important factor that determines ionic strength on the cells and thereby the viability of cells post electroporation is the electroporation buffer. It is recommended to maintain hypo-osmolar conditions during electroporation since it enables easier and controlled electroporation [23]. However, some sources recommend iso-osmolar conditions to promote efficient DNA uptake and cell viability [18].

Whereas the gene delivery is the primary aim and protein expression being the ultimate aim of the transfection, viability is critical in terms of maintaining critical seeding density post electroporation. Though there are recent advances in electroporation technique, like micro- and nano-electroporation, such novel strategies have not yet been demonstrated to supersede the basic cuvette-style electroporation [3]. Hence, in spite of being a well-established technique, there is still a great potential to enhance the square wave electroporation outcome. Also, the rational cell type-dependent approach of electroporation, paves the way for getting additional insights into physical prerequisites for optimum transfection and better electroporation outcomes [24]. Hence, we hypothesized that such improved transfection performances can be obtained with selective interventions at critical steps in the process like choice of electroporation buffer, altering pulse parameters, and type of the cuvettes. The hypothesis was drawn by considering the already established concept of Maxwell-Wagner polarization, a key parameter for electroporation, which is an induced transmembrane voltage generated by an external electric field due to the variations in electrical properties of cell membrane, cytoplasm, and external medium [25]. Here, the transient expression was assessed, but a high initial transfer is of course a prerequisite for a stable long-term transformation.

## Results

### Opti-MEM with GlutaMax improves viability of cells during electroporation

**Fig. 1:**
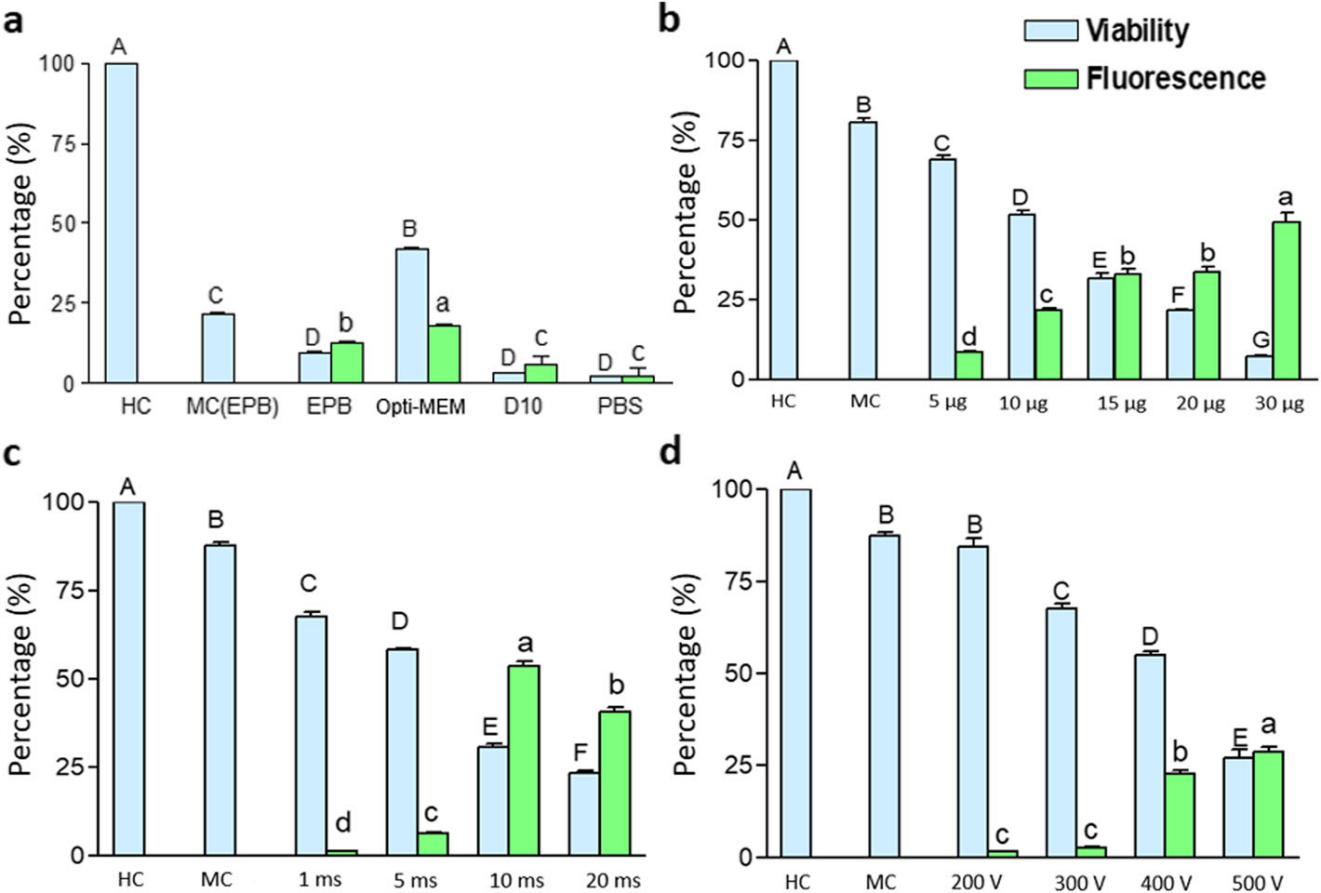
Critical parameters for the transfection of BFF. **a** Effect of electroporation medium (other electroporation conditions: 4 mm cuvette, 1 pulse, 400 V, 10 milliseconds, 5 μg DNA, room temperature handling). HC, handling control; MC, mock control; EPB, Biorad (Gene Pulser) electroporation buffer; Opti-MEM, ThermoFisher medium with GlutaMax; D10, fibroblast cell culture medium; PBS, phosphate buffered saline. **b** Influence of DNA concentration on transfection efficiency (other electroporation conditions: 4 mm cuvette, 1 pulse, 300 V, 10 milliseconds, opti-MEM as electroporation buffer, room temperature handling). **c** Influence of pulse duration on transfection efficiency (other electroporation conditions: 4 mm cuvette, 1 pulse, 400 V, 10 μg DNA, opti-MEM as electroporation buffer, room temperature handling). **d** Influence of pulse voltage on transfection efficiency (other electroporation conditions: 4 mm cuvette, 1 pulse, 5 μg DNA, 10 milliseconds, opti-MEM as electroporation buffer, room temperature handling). All the values are indicated by mean ± SEM. Means bearing different superscripts in upper case differ significantly in terms of viability, and those bearing different superscripts in lower case differ significantly in terms of fluorescence. Number of replicates = 3

Four different electroporation buffers, Gene Pulser electroporation buffer (Bio-Rad), Opti-MEM (ThermoFisher), phosphate buffered saline (PBS), and D10 cell culture medium, were tested. The use of Opti-MEM was found to result in the best combination of of cell viability and ratio of Venus-positive cells (Fig. 1a; Fig. S1, S2). Electroporation of bovine fetal fibroblasts with Opti-MEM resulted in 40% vital cells, of which almost half were Venus-positive. The pulse conditions used in this experiment are 400 V pulse for 10 milliseconds in 4 mm cuvettes using 5 μg DNA and the entire procedure was performed at room temperature.

### Concentration of DNA is inversely proportional to viability

Various plasmid concentrations were tested, i.e. 5, 10, 15, 20, 30 μg per 100 μl electroporation buffer. With increasing concentrations of DNA, the viability of bovine fibroblasts was decreasing (Fig. 1b; Fig. S3). Each of the higher concentration tested had significantly lesser viability as compared to the preceding lower concentration of DNA. The optimal DNA amount with respect to viability and Venus-positive cells was determined to be 10 μg of plasmid, using the parameters single pulse of 300 V for 10 milliseconds in 4 mm cuvettes with Opti-MEM as electroporation buffer. The entire procedure was performed at room temperature.

### Optimal pulse duration greatly determines the transfection efficiency

**Fig. 2:**
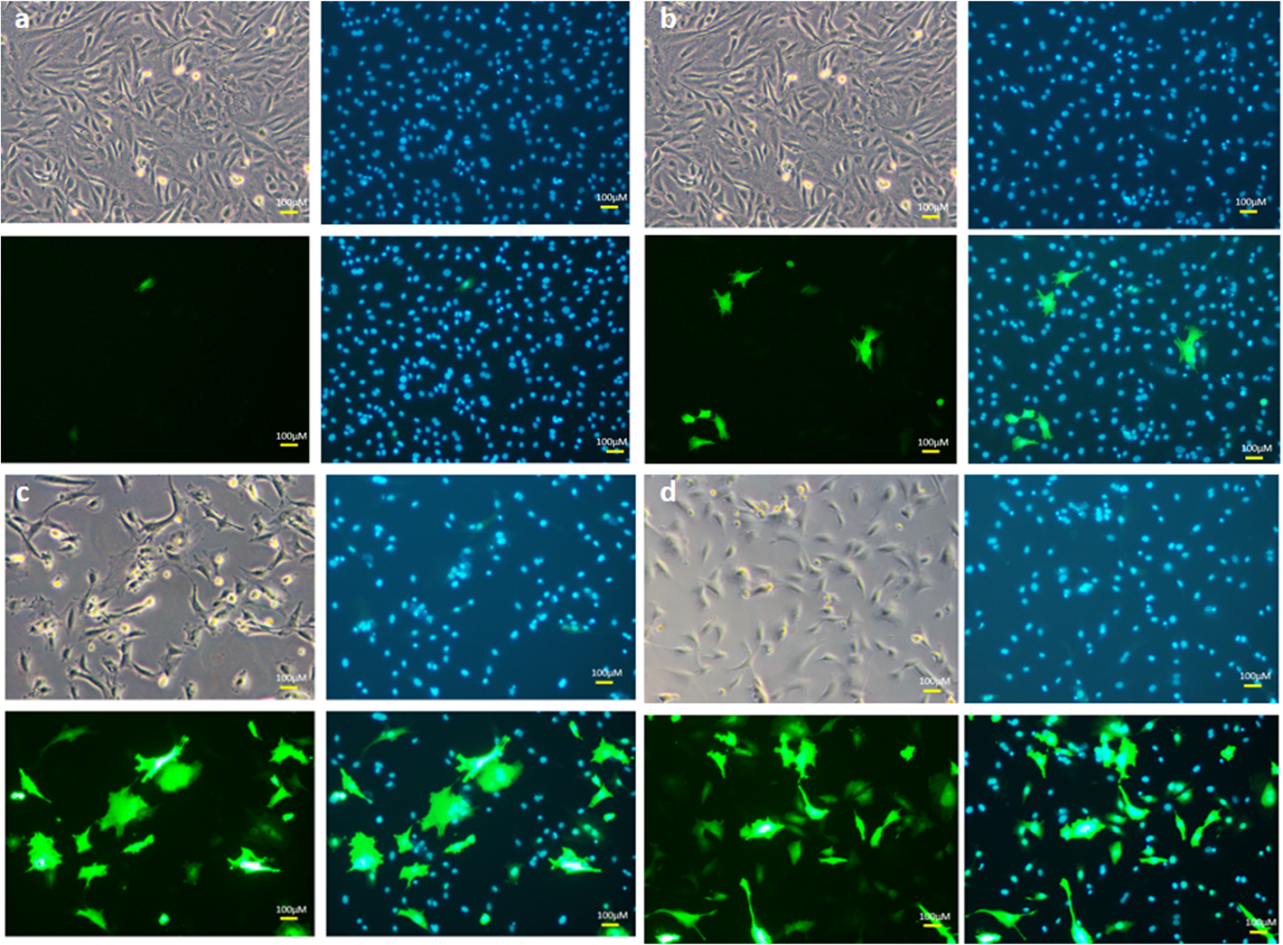
Transfection efficiency in bovine fetal fibroblasts with varying pulse durations. **a** 1 ms, **b** 5 ms, **c** 10 ms, **d** 20 ms. Brightfield, Hoechst 33342-stained nuclei, Venus fluorescent cells and merged images are shown. Number of replicates = 3. (other electroporation conditions: 4 mm cuvette, 1 pulse, 400 V, 10 μg DNA, opti-MEM as electroporation buffer, room temperature handling)

With respect to various pulse durations tested, i.e. 1, 5, 10, 20, 30 milliseconds, we observed that an increase in pulse durations resulted in significant decreased cell viabilities. With a 10 millisecond pulse the viability was found to be about 30% (Fig. 1c; Fig. 2) and the best ratio of Venus-positive cells was obtained. The other pulse conditions were 400 V of single pulse using 10 μg of DNA with Opti-MEM as electroporation buffer in 4 mm cuvettes. The entire procedure was performed at room temperature.

### Optimization of the pulse voltage

With respect to the pulse voltages of 200, 300, 400, 500 V were tested. Increasing pulse voltages correlated directly with an increased ratio of Venus-positive cells, but inversely with cell viability. The optimum pulse voltage was found to be 400 V (Fig. 1d; Fig. S4). The other pulse conditions were single pulse of 10 milliseconds using 5 μg of DNA in 4 mm cuvettes with Opti-MEM as electroporation buffer. The entire procedure was performed at room temperature.

### Pulse number has no significant improvement on transfection efficiency

Next it was tested, if the application of more than one pulse improved the transfection efficiency. However, no significant improvement of the transfection efficiency was found if the number of pluses were increased (Table 1). For multiple pulses the pulse interval was kept constant at 1 ms. The other pulse conditions were 200 V with 4 μg of DNA in 2 mm cuvettes. The entire procedure was performed at room temperature in Opti-MEM as electroporation buffer.

**Table 1:** Effect of pulse number on transfection efficiency

No of pulses	Viability (%)	Fluorescence (%)
1	65.0 ± 0.89^a^	7.2 ± 0.20 ^a^
2	60.6 ± 0.92^b^	6.2 ± 0.18 ^b^
3	64.8 ± 0.73^a^	7.8 ± 0.13 ^a^

Other pulse conditions: 200 V; pulse interval, 1 ms; pulse duration, 10 ms; 2 mm cuvette; 4 μg DNA; number of replicates =3. The electroporation buffer was BioRad electroporation buffer

Different superscript letters indicate significant difference (*P* < 0.05)

### Precooling the cuvettes drastically reduced the transfection efficiency

Previous studies proved that some of the cells are very sensitive to alterations in handling temperatures [26]. To assess the effect of the temperature, bovine fibroblasts were electroporated in parallel at 4 °C (cuvettes pre-cooled on wet ice for 15 min) and at room temperature. Unexpectedly, precooling of cuvettes significantly reduced viability and transfection efficiency (Table 2). The other pulse conditions were 200 V of a single pulse using 4 μg of DNA in 2 mm cuvettes with Opti-MEM as electroporation buffer. Apart from precooling the entire procedure was performed at room temperature.

**Table 2:** Effect of temperature on transfection efficiency

Temperature	Viability (%)	Fluorescence (%)
Room temperature	13.4 ± 0.51 ^a^	7.3 ± 0.20 ^a^
4 °C	2.4 ± 0.24 ^b^	5.8 ± 0.15 ^b^

Other pulse conditions: 300 V; 1 pulse; pulse duration, 10 ms; 2 mm cuvette; 4 μg DNA; number of replicates = 3. The electroporation buffer was Opti-MEM with GlutaMax

Different superscript letters indicate significant difference (*P* < 0.05)

### Cuvettes with higher electrode gap have better transfection efficiency

Next, the effect of the electrode distance on the transfection efficiency was studied. Therefore either 2 mm or 4 mm cuvettes were used. We observed that 4 mm cuvettes had significantly better transfection efficiencies than 2 mm cuvettes (Table 3). The other pulse conditions were 200 V of a single pulse of 10 milliseconds duration with 4 μg DNA with Opti-MEM as electroporation buffer.

**Table 3:** Effect of cuvette type on transfection efficiency

Cuvette type	Viability (%)	Fluorescent cells of vital cells (%)
2 mm	22.8 ± 1.1	17.2 ± 0.75
4 mm	61.0 ± 0.7	23.8 ± 0.61

Other pulse conditions: 200 V; 1 pulse; pulse duration, 10 ms; 4 μg DNA; number of replicates = 3. The electroporation buffer was Opti-MEM with GlutaMax

## Discussion

Electroporation is a common intracellular delivery method that has been reported to be utilized even for transfecting CRISPR/Cas9 compounds [27]. It has been proven that if the electric field pulse has the appropriate characteristics, the “electroporated” cells recover with normal functionality [28]. Some reports state the mechanism of delivery of large molecules like nucleic acids entirely depend upon electrophoretic forces provided during the pulse [29]. Here, it was found that the transfection rates of bovine fetal fibroblasts critically depend on the selected square-wave pulse conditions and the used electroporation buffer. Optimized transfection rates could be achieved not only by fine-tuning the pulse conditions, but also by altering other critical parameters. The optimal pulse conditions were a single 10 ms square-wave pulse of 400 V in a 4 mm cuvette. Other critical parameters were i) the electroporation buffer ii) the electroporation temperature, iii) and the amount of plasmid DNA.

We observed that the electroporation buffer in which the plasmid DNA was delivered by electroporation played the most critical role. Among the four electroporation buffers, Opti-MEM proved to be the best as it gave better transfection efficiency and superior cell viability in comparison to commercial Bio-Rad buffer, PBS and D10 medium. Opti-MEM is a serum-reduced medium and it contains insulin, transferrin, thymidine, hypoxanthine, and trace elements (https://www.thermofisher.com/order/catalog/product/31985070). It also contains GlutaMAX supplement, which is a dipeptide, L-alanine-L-glutamine, and earlier studies have proven that polymers like poly-L-glutamate can increase efficacy and reduce toxicity of electroporation [30]. Opti-MEM ensures favorable Maxwell-Wagner polarization conditions, a prerequisite for better electroporation outcomes, since previous studies proved external medium to be critical factor for generation of induced transmembrane voltages [25]. Whereas the D10 medium is used for culturing the cells routinely, its use as electroporation buffer resulted in poor transfection efficiencies, which could be attributed to negative effects of serum on transfection, since serum proteins are proven to compete with transfection vehicles/vectors for entry into cells [31]. With regard to PBS, our results were in disagreement with the study conducted by Kang et al. [32] on primary endothelial cells who reported comparable transfection efficiencies with tolerable decrease in viability. We could neither observe comparable viability nor transfection efficiency as compared to Opti-MEM and Bio-Rad buffer indicating overall poor Maxwell-Wagner polarization conditions.

Another important factor in electroporation is the amount of DNA added to the cell suspension. We tested concentrations ranging from 5 μg to 30 μg per 100 μl electroporation volume. We noticed that with increased plasmid concentrations, the transfection efficiency is increased, however at the same time the cell viability is reduced. This finding is in agreement with a previous study [33]. Here, the optimal concentration of plasmid DNA for the transfection of BFFs was determined to be 10 μg per 100 μl. The same concentration was earlier proposed even for other types of difficult to transfect cells, like sperms [34] and Eol-1 cells [35]. Though higher concentrations of DNA increased transfection rate it caused greater cell death owing to the plasmid dependent changes in osmolality of cell suspensions. Typically, mammalian cells showed better transfection rates in hypotonic solutions [36].

The other two conditions that determine the transfection efficiency are voltage and duration of exposure to a particular voltage. Whereas either microsecond or millisecond pulses can be used in electroporation, for transfection of macromolecules, like DNA, the millisecond pulse is desirable for increased uptake [37]. Here, the 10 millisecond pulse was found to be optimal in terms of viability and transfection rate; extended exposure time resulted in drastically decreased viability that might be attributed to excessive heat generated during the pulse as previous studies reported that conventional cuvette type electroporation can cause heat generation [38]. Also, with longer duration of pulse, increased number of pores may result in a condition where pores may coalesce leading to cell death [39], whereas the lesser transfection efficiency observed in short duration pulse can be attributed to formation of only transient pores, which are not stable enough for the entry of molecules [25]. Previous studies have shown mixed results with respect to multiple pulse applications and reported complex dependencies [40]. The complexity depends upon conditions of treatment and their associated effects on cells like heating, pore size, leakiness, and resealing of cell membrane [39, 40]. Hence in our experiments, the complex results observed can be attributed to pulse-interval associated resealing of membrane and corresponding alterations in fractional area of pores, since previous studies reported decrease in fractional area of pores with multiple pulsations [40]. It was earlier reported that at a constant number of pulses, it is the time between pulses that strongly affects transfer of molecules [1]. Previous studies also reported that varying pulse intervals can lead to situations like entry of Ca^+ 2^ ions in the cells which at a particular concentration make the cells vulnerable [40]. With respect to voltage, 400 V pulse settings were determined to be ideal for electroporation when dealing with 4 mm cuvettes. Though the 500 V with 4 mm cuvettes produce same field strength (1.25 kV/cm) as 250 V with 2 mm cuvettes, the efficiency of electroporation was better in 4 mm cuvettes probably owing to the greater distance of cells from electrodes, that prevents contact of extensive number of cells with electrodes. Previous studies proved that cell distance from electrodes is one of the critical parameters that affects electroporation outcomes [41]. Also, it is only at high electric field strengths that cell membrane progressively become less resistive [42]. The suitability of the high-voltage and low-voltage (HVLV) puls method [43] for bovine primary fibroblasts nessitates future research.

The drastic decrease in transfection efficiency and viability with pre-cooling of cuvettes could be attributed to temperature induced alterations in membrane fluidity. It is well established that the ability to respond to thermal changes is one of the important properties of membranes, playing an essential role in maintaining fluidity [44]. Another reason attributed for lower transfection efficiency due to pre-cooling of cuvettes might be that lower temperatures are reported to increase the electric field strength and retarding the kinetics of cell membrane re-sealing [3]. It was shown before that not only membrane fluidity, but membrane domain changes determine temperature induced alterations on electroporation effects [28].

## Conclusions

It can be concluded that improved transfection efficiency can be achieved in difficult to transfect cells like bovine primary fibroblasts by testing and fine-tuning various parameters. The most critical parameters are the pulse conditions, electroporation buffer, electroporation temperature and the amount of plasmid DNA.

## Methods

### Bovine fetal fibroblasts culture

Bovine fetal fibroblasts were derived and cultured as described before [45]. In brief, bovine fetuses (~ 2–3 months of gestation) were collected from a local slaughterhouse, rinsed in 80% ethanol and the skin tissue was minced in ice-cold PBS supplemented with antibiotics. Minced tissue pieces (< 1 mm^3^) were transferred into a 6-well cell culture plate, a cover slip was placed onto the tissue pieces, 4 ml Dulbecco’s Modified Eagle Medium with 10% serum (D10) was added and cultures were incubated at 37 °C, 5% CO_2_ in a humidified incubator (Thermo Scientific, #50116047). The primary fibroblasts were splitted when they reached a confluence of 60–80% by EDTA/trypsin (GE Healthcare, #L11–003)treatment, and cells of passage 2 were resuspended in a cryopreservation medium (90% cell culture medium and 10% dimethyl sulfoxide (Sigma-Aldrich, #322415-100ML), and frozen in aliquots at − 80 °C.

### Reporter construct

The pT2-RMCE-Venus (6301 bp) encoding a ubiquitously expressed Venus fluorophore reporter [46] was used as an indicator for the assessment of transfection efficiency. The Venus cDNA is driven by a CAGGS promoter, a hybrid construct consisting of the cytomegalovirus (CMV) enhancer fused to the chicken beta-actin promoter. The plasmid was confirmed by sequencing.

### Electroporation of BFFs

About 3 million fibroblasts cultured in a T75 flask (Sarstedt AG & Co. KG, #83.3911.002) were trypsinized, pelleted by a gentle centrifugation (Thermoscientific #75004250), and resuspended in 900 μL of electroporation buffer. One hundred microliter (equivalent to 0.3 × 10^6^ cells) aliquots were mixed with the plasmid DNA and then transferred into electroporation cuvettes (2 mm (Cell Projects, #EP-102) or 4 mm (Cell Projects, #EP-104). A Bio-Rad Gene Pulser Xcell (Gene Pulser Xcell Eukaryotic System, Order no:1652661) was used for pulsing, the following parameters could be set for square wave pulses: voltage, pulse duration, plus number, and interval between pulses. The pulses were recorded online, and typically a droop of 2–3% of the set voltage was measured. For every experiment a handling control (100 μL of cell suspension seeded in one of the wells of a 6-well plate) and a mock control (100 μL of cell suspension electroporated without plasmid) were performed. After electroporation, the cells were transferred into one well of a 6-well plate (Sarstedt AG & Co. KG, #83.3920.300), culture medium was added and the plate was placed in an incubator. The next day, cells were observed for viability and fluorescence. For the sub-experiment of testing the efficiency of electroporation buffer on electroporation outcomes, flow cytometry measurements were done to confirm the microscopic evaluations. The conditions tested with respect to transfection efficiency of electroporation, were type of electroporation buffer, varying concentrations of DNA, electroporation pulse parameters, like voltage, number and duration of pulse, type and temperature of cuvettes. The used electroporation buffers are Gene Pulser Electroporation Buffer (Biorad #165–2677), Opti-MEM with Reduced Serum Medium with GlutaMax Supplement (ThermoFisher, #51985–026), phosphate buffered saline (Dulbecco’s PBS; Sigma-Aldrich, D5652) and D10 culture medium. All electroporation steps were carried out at room temperature conditions unless otherwise stated specifically.

### Analysis of transfected cells

Cells were observed for viability and fluorescence 24 h post-transfection. The culture media was changed to get rid of dead cells and new media was added. For each of the wells 2 μL of Hoechst 33342 (Sigma Aldrich, # 875756–97-1) (1 mg/ml) was added to stain the nuclei. The cells were then observed under a Zeiss Axiovert 35 M microscope equipped with fluorescence optics for UV (320–360 nm), blue (450–490 nm) and red (550–580 nm) fluorescence excitation. Alternatively, images were obtained by an Olympus BX 60 (Olympus, Hamburg, Germany) fluorescence microscope equipped with a high resolution digital camera (Olympus DP71). The viability of cells in treatment groups was determined by the formula: Viability (%) = (No. of cells in treatment group / No. of cells in Handling control) × 0.01.

Fluorescence percentage was calculated within each treatment group as number of Venus-positive cells/total number of viable cells.

### Flow cytometry

For the flow cytometry analysis, the transfected cells were allowed to proliferate for 5 days to obtain sufficient cells numbers, a prerequisite for flow analysis. In cases of poor proliferation less than 10,000 events were counted by flow cytometry. The FACS analysis was performed using a MACSQuant Analyzers (Merck KGaA, Darmstadt, Germany). Prior to analysis, cells were washed and suspended in FACS buffer (PBS), containing 1% FBS, and cell concentrations were adjusted to ~ 5 × 10^5^ cells/mL. Data were analyzed using Guava Soft 3.1 and FlowJoX (Tree Star, Ashland, OR, USA).

### Data analysis

All data were expressed as mean ± SEM. Statistical analyses were performed using the SPSS 17.0 statistical software package. One-way ANOVA was used with sampling period as fixed factor. Pair wise comparisons (or post hoc test) were based using the T-method (Tukey’s honestly significant difference method). The minimum significant range of confidence was evaluated at 0.05 level.

## Supplementary information

**Additional file 1**: **Figure S1.** Analyzing the effect of electroporation buffer on transfection efficiency using flow cytometry. **Figure S2.** Analyzing the effect of electroporation buffer on transfection efficiency. **Figure S3.** Analyzing the effect of DNA concentration on transfection efficiency. **Figure S4.** Analyzing the effect of pulse strength on transfection efficiency.

## Data Availability

Data and materials are available upon request from the corresponding author.

## Abbreviations

BFF: Bovine fetal fibroblast
Crispr/Cas9: Clustered regularly interspaced short palindromic repeats-associated protein 9
EDTA: Ethylenediaminetetraacetic acid
FACS: Fluorescence activated cell sorting
PBS: Phosphate buffered saline
TALEN: Transcription activator-like effector nucleases
ZNF: Zinc finger nuclease
