# Supplementary material for: Systematic optimization of square-wave electroporation conditions for bovine primary fibroblasts
Source: BMC Mol Cell Biol. 2020 Feb 28;21:9. doi: 10.1186/s12860-020-00254-5 (PMC7049184; doi:10.1186/s12860-020-00254-5)
Supplement: Supplementary file 1 — Additional file 1: Figure S1. Analyzing the effect of electroporation buffer on transfection efficiency using flow cytometry. Figure S2. Analyzing the effect of electroporation buffer on transfection efficiency. Figure S3. Analyzing the effect of DNA concentration on transfection efficiency. Figure S4. Analyzing the effect of pulse strength on transfection efficiency. [file 12860_2020_254_MOESM1_ESM.docx]

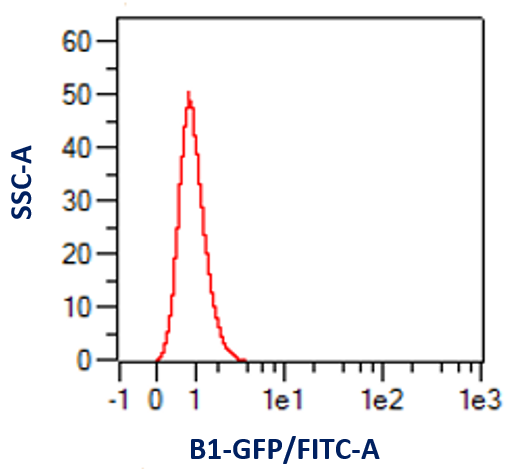

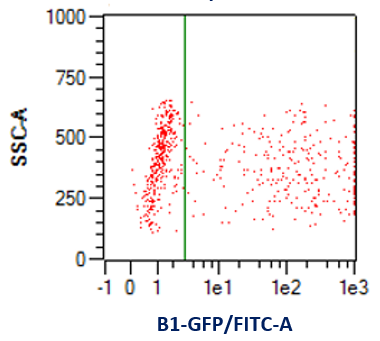


**a**

**b**

**c**


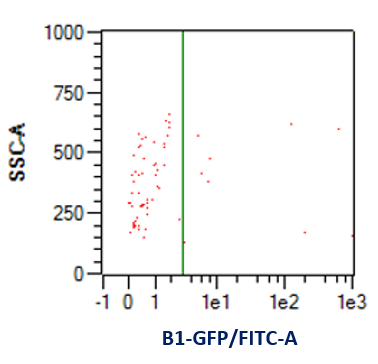


Fig. S1: **Analyzing the effect of electroporation buffer on transfection efficiency using flow cytometry. a**) Non-transfected cells as negative control (Number of cells analyzed: 14,598). b) Cells transfected in the carrier medium of Opti-MEM had 40.33% transfection rate (Number of cells analyzed: 9381), whereas with c) commercial Biorad Buffer it was only 12.31% (Number of cells analyzed: 1729).

**b**

**a**


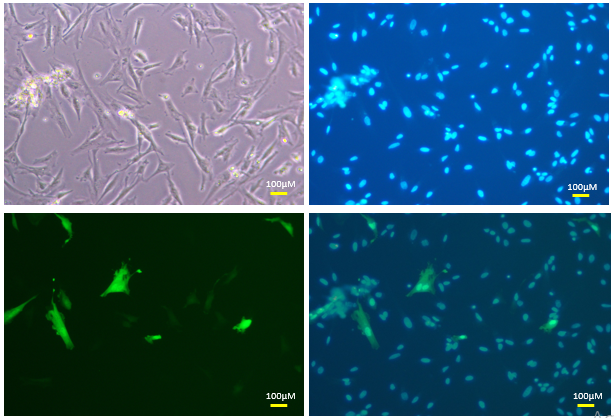


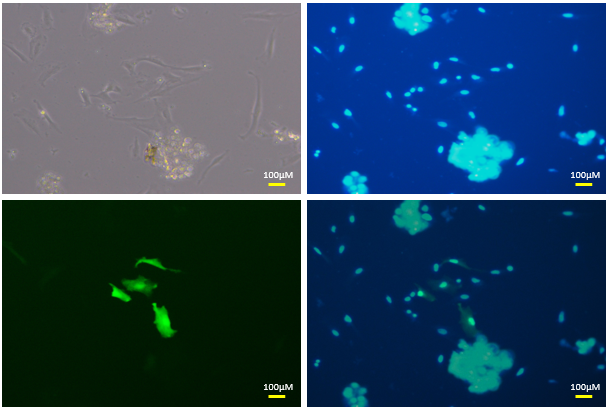


**c**


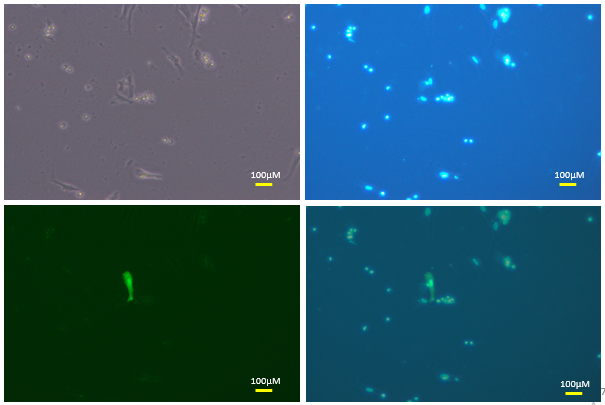


Fig. S2: **Analyzing the effect of electroporation buffer on transfection efficiency**

Comparison of transfection efficiency in bovine embryonic fibroblasts with different electroporation buffers, Biorad electroporation buffer (a), Opti-MEM with GlutaMax (b), PBS (c). Opti-MEM has better transfection efficiency than other two solutions. In each of the figure sub-sets, the four images starting from the left top clockwise are brightfield, Hoechst 33342-stained nuclei, merge of Venus and Hoechst fluorescence, and Venus fluorescence, respectively. (Other pulse conditions: square wave, 4 mm cuvette, 1 pulse, 400V, 10 milliseconds, 5 µg DNA, room temperature handling).

**b**

**a**


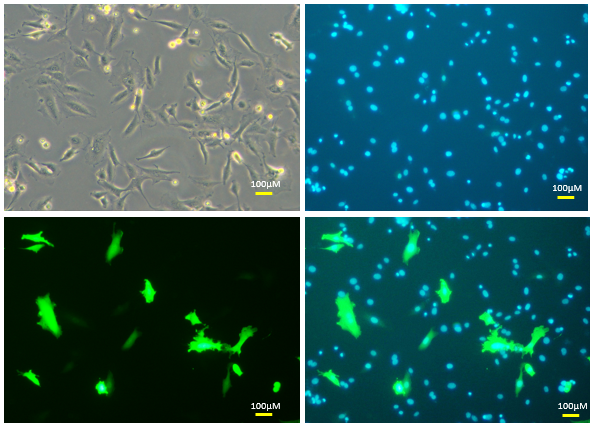


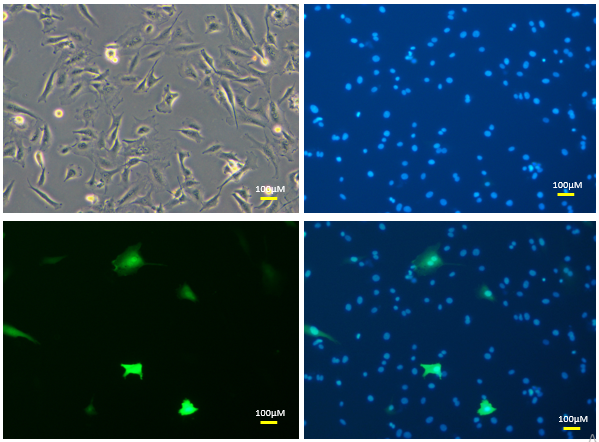


**d**

**c**


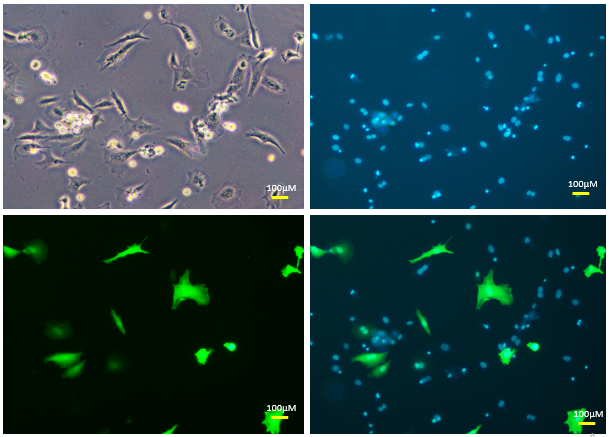


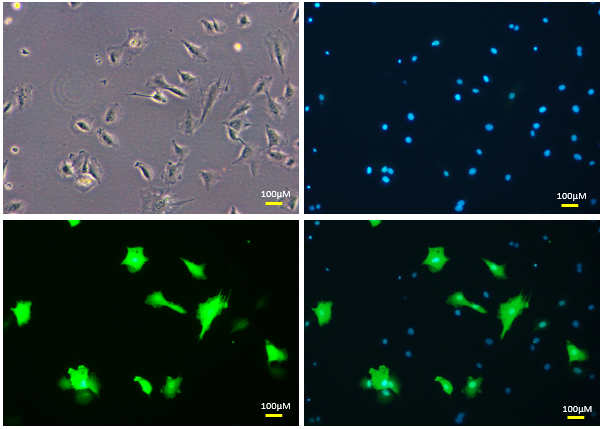


Fig. S3: **Analyzing the effect of DNA concentration on transfection efficiency**

Comparison of transfection efficiency in bovine embryonic fibroblasts with varying DNA concentrations i.e., (a) 5µg (b) 10µg (c) 15µg (d) 20µg. In each of the figure sub-sets, the four images starting from the left top clockwise are brightfield, Hoechst 33342-stained nuclei, merge of Venus and Hoechst fluorescence, Venus fluorescence, respectively. (other pulse conditions: 4 mm cuvette, 1 pulse, 300V, 10 milliseconds, opti-MEM as electroporation buffer, room temperature handling).

**b**

**a**


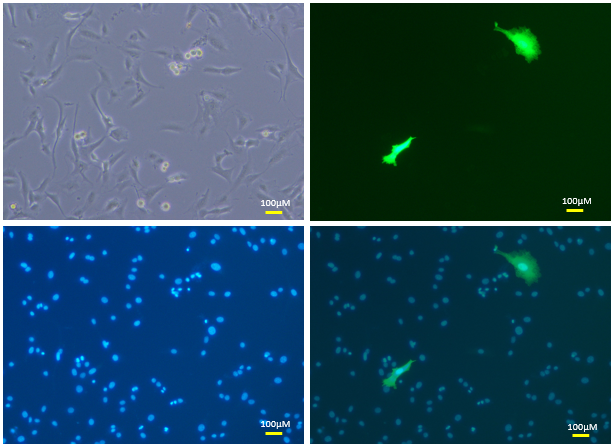


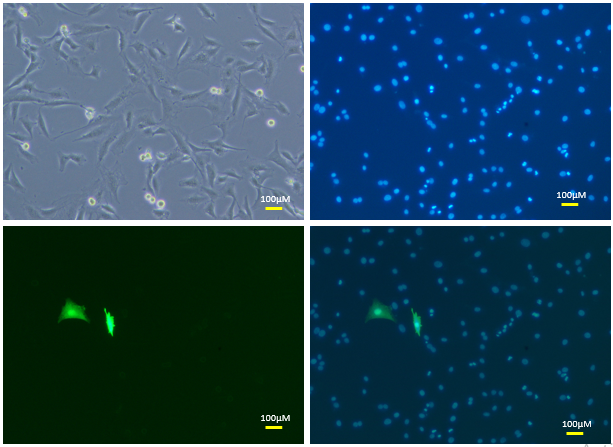


**d**

**c**


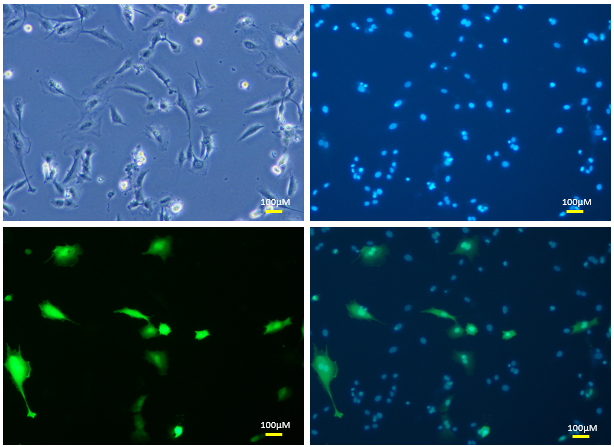


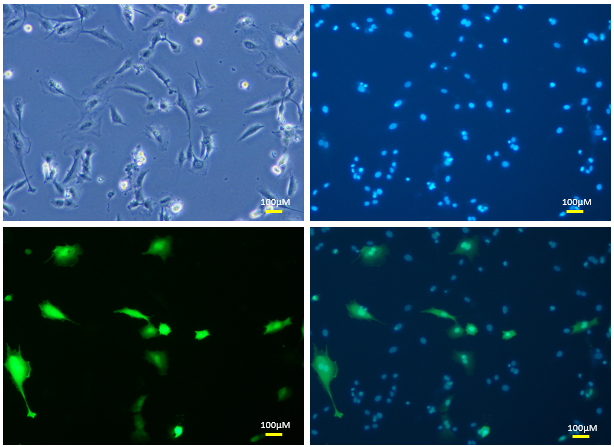


Fig. S4: **Analyzing the effect of pulse strength on transfection efficiency**

Comparison of transfection efficiency in bovine embryonic fibroblasts with varying pulse voltages i.e., (a) 200V (b) 300V (c) 400V (d) 500V. In each of the figure sub-sets, the four images starting from the left top clockwise are brightfield, Hoechst 33342-stained nuclei, merge of Venus and Hoechst fluorescence, and Venus fluorescence, respectively. (other pulse conditions: 4 mm cuvette, 1 pulse, 5 µg DNA, 10 milliseconds, opti-MEM as electroporation buffer, room temperature handling).
